# Exenatide regulates Th17/Treg balance via PI3K/Akt/FoxO1 pathway in db/db mice

**DOI:** 10.1186/s10020-022-00574-6

**Published:** 2022-12-03

**Authors:** Qinqin Xu, Xiaoling Zhang, Tao Li, Shiying Shao

**Affiliations:** 1 Division of Endocrinology, Tongji Hospital, Huazhong University of Science and Technology, Jiefang Road 1095, Wuhan, 430030 Hubei Province People’s Republic of China; 2 Branch of National Clinical Research Center for Metabolic Diseases, Hubei, People’s Republic of China; 3 Division of Ophthalmology, Tongji Hospital, Huazhong University of Science and Technology, Jiefang Road 1095, Wuhan, 430030 Hubei Province People’s Republic of China

**Keywords:** Type 2 diabetes mellitus, T helper 17 cell, T regulatory cell, Exenatide, Forkhead box O1

## Abstract

**Background:**

The T helper 17 (Th17)/T regulatory (Treg) cell imbalance is involved in the course of obesity and type 2 diabetes mellitus (T2DM). In the current study, the exact role of glucagon-like peptide-1 receptor agonist (GLP-1RA) exenatide on regulating the Th17/Treg balance and the underlying molecular mechanisms are investigated in obese diabetic mice model.

**Methods:**

Metabolic parameters were monitored in db/db mice treated with/without exenatide during 8-week study period. The frequencies of Th17 and Treg cells from peripheral blood and pancreas in db/db mice were assessed. The phosphoinositide 3-kinase (PI3K)/protein kinase B (Akt)/Forkhead box O1 (FoxO1) pathway in Th17 and Treg cells from the spleens of male C57BL/6J mice was detected by western blotting. In addition, the expression of glucagon-like peptide-1 receptor (GLP-1R) in peripheral blood mononuclear cells (PBMCs) of male C57BL/6J mice was analyzed.

**Results:**

Exenatide treatment improved β-cell function and insulitis in addition to glucose, insulin sensitivity and weight. Increased Th17 and decreased Treg cells in peripheral blood were present as diabetes progressed while exenatide corrected this imbalance. Progressive IL-17 + T cell infiltration of pancreatic islets was alleviated by exenatide intervention. In vitro study showed no significant difference in the level of GLP-1R expression in PBMCs between control and palmitate (PA) groups. In addition, PA could promote Th17 but suppress Treg differentiation along with down-regulating the phosphorylation of PI3K/Akt/FoxO1, which was reversed by exenatide intervention. FoxO1 inhibitor AS1842856 could abrogate all these effects of exenatide against lipid stress.

**Conclusions:**

Exenatide could restore systemic Th17/Treg balance via regulating FoxO1 pathway with the progression of diabetes in db/db mice. The protection of pancreatic β-cell function may be partially mediated by inhibiting Th17 cell infiltration into pancreatic islets, and the resultant alleviation of islet inflammation.

**Supplementary Information:**

The online version contains supplementary material available at 10.1186/s10020-022-00574-6.

## Background

The prevalence of type 2 diabetes mellitus (T2DM) is rising at a considerable rate, with a global burden on public health. The pathophysiological mechanisms of T2DM are complex, including environmental factors, genetic defects, glucotoxicity, lipotoxicity, endoplasmic reticulum (ER) stress, and oxidative responses (DeFronzo et al. 2015). Recently, it is recognized that a chronic low-grade inflammation and activated immune system also play essential role in the insulin resistance (Lee et al. 2018; Zeyda et al. 2009). This chronic inflammatory state occurs in pancreatic islets as well, leading to β-cell dysfunction (Ying et al. 2020). However, the molecular mechanisms leading to islet inflammation are incompletely identified. The understanding of these pathophysiological abnormalities provides an expansion of potential targets for T2DM treatment.

Interleukin (IL)-17-producing CD4 + T helper (Th17) cells are crucial for the chronic inflammation and autoimmunity (Singh et al. 2014) while regulatory T (Treg) cells can suppress inflammatory responses and maintain peripheral tolerance (Sakaguchi et al. 2008). The imbalance of these two lymphocytes has been identified to be involved in the pathogenesis of type 1 diabetes mellitus (T1DM) (Fabbri et al. 2019). Furthermore, we recently described the potential role of Th17 and Treg cells in obesity and T2DM (Wang et al. 2018). It is found that the proportion of Th17 cells was higher in obese or T2DM patients accompanied by elevated level of inflammatory cytokines and the deterioration of glucose homeostasis (Dalmas et al. 2014; Ip et al. 2016). In contrast, Tregs and related cytokines IL-10 and transforming growth factor-β (TGF-β) were precipitously decreased in newly diagnosed T2DM patients along with a negative correlation between TGF-β and homeostasis model assessment of insulin resistance (HOMA-IR) (Yuan et al. 2017). In addition, adoptive transfer of Tregs was found to be associated with reversal of insulin resistance in leptin receptor-deficient db/db mice (Eller et al. 2011), a model animal for T2DM characterized by hyperphagia, obesity, hyperinsulinemia and hyperglycemia (King 2012). Thus, we speculate that Th17/Treg imbalance may be involved in immune responses that contribute to both islet and systemic inflammation under the context of obesity and T2DM. Regulation of their balance might exert a beneficial effect on the treatment of T2DM.

Glucagon-like peptide-1 (GLP-1) is an incretin hormone secreted from the gastrointestinal tract and functions to lower glucose concentrations by augmenting insulin secretion and suppressing glucagon release (Drucker 2018). To exert its physiological effects, GLP-1 binds specifically to GLP-1 receptor (GLP-1R), resulting in the cyclic adenosine monophosphate (cAMP)-dependent activation of second messenger pathways (Lamont et al. 2012; Shao et al. 2022). Of note, emerging evidences have indicated a critical role for GLP-1 in modulating innate immunity and inflammation. This anti-inflammatory property might be associated with both the indirect metabolic improvement (Cechin et al. 2012; Lee et al. 2012) and direct regulation of systemic proinflammatory cytokines and infiltration of immune cells in specific tissues, which has been discussed in our previous work (Shao et al. 2022).

There are a variety of immune cells that express GLP-1Rs including CD4 + T cells, CD8 + T cells, macrophages, and B cells (Hadjiyanni et al. 2010; Hogan et al. 2011; Stahle et al. 2021; Tanaka et al. 2016). These findings suggest that the potential effects of GLP-1 in immunomodulation could be independent of glucose control and weight loss. A recent study verified that GLP-1 receptor agonist (GLP-1RA) could improve albuminuria by inhibiting CD4 + T cell proliferation in a GLP-1R-dependent manner in a T-cell–mediated murine model of nephrotoxic serum nephritis (NTS) (Moschovaki Filippidou et al. 2020). Charpentier et al. demonstrated that GLP-1RA liraglutide could regulate intestinal immune system via increasing the frequency of Treg cells in diet-induced dysmetabolic mice (Charpentier et al. 2021). In spite of these advances, whether GLP-1 and its agonists could inhibit islet inflammation and thus improve β-cell function through regulating Th17/Treg balance under the context of T2DM remains largely undefined.

Transcription factor forkhead-box O1 (FoxO1), a member of FoxO family, is modulated mainly by the phosphoinositide 3-kinase (PI3K)/protein kinase B (Akt) signal (Laine et al. 2015; Ouyang et al. 2010). FoxO1 has been reported to have a crucial role for T cell development and might provide a link between metabolism and immunity (Hedrick et al. 2012). This transcription factor is a negative regulator of Th17 program by inhibiting retinoid-related orphan receptor gamma-t (ROR-γt) activity (Laine et al. 2015); but plays a positive role in Treg differentiation and function (Ouyang et al. 2010, 2012). In addition, our previous study demonstrated that liraglutide could exert protective effects on β-cell function under lipotoxic stress via PI3K/Akt/FoxO1 pathway (Shao et al. 2014). It would be significant to figure out whether PI3K/Akt/FoxO1 takes participate in GLP-1RA-regulated Th17/Treg balance.

In this study, it is aimed to identify the critical role for Th17 and Treg in islet inflammation under the context of obesity and T2DM. Exenatide was synthetically developed as a recombinant structure of exendin-4 and was the first GLP-1RA for the treatment of T2DM (Mikhail 2006). We also investigated the exact role of short-acting exenatide on Th17/Treg equilibrium in db/db mice and the potential mechanisms with FoxO1 involved.

## Materials and methods

### Animals and groups

Sixteen 4-week-old male db/db mice were randomly divided into exenatide (n = 8) or control group (n = 8) and maintained in a specific-pathogen free, temperature-controlled environment under 12 h light/dark cycles. Mice in exenatide group received subcutaneous short-acting exenatide (Byetta, Baxter Pharmaceutical Solutions LLC., Indiana, USA) administrations (200 µg/kg body weight) (Bameri et al. 2021; Tatarkiewicz et al. 2013). Control mice received an equal volume of saline solution. No insulin or other oral hypoglycemic agents were given during the experiment to prevent the interference of confounding factors. After 8-week treatment, animals were sacrificed and samples were collected. All animal care and experimental protocols were approved by the Institutional Animal Care and Use Committee of Tongji Medical College.

### Glucose tolerance and insulin measurement

Body weight and fasting glucose level were regularly measured every 3 days. Oral glucose tolerance test (OGTT) and fasting insulin were detected at week 0 and week 8, respectively. For OGTT, body weight was determined after 12 h overnight fasting and then mice were administrated by gavage with 2 g/kg body weight glucose solution. 100 µL blood samples were collected before and 30, 60, 90, 120 and 180 min after the gavage via capillary pipette from caudal vein and placed into EDTA-treated tube for glucose measurement. Serum fasting insulin was assayed using Mouse Insulin ELISA Kit (#CSB-E05071m, Cusabio, China). HOMA-IR and HOMA of β-cell function (HOMA-β) were calculated as follows: HOMA-IR = Glucose (mmol/L) × Insulin (mU/L)/22.5; HOMA-β = 20 × Insulin (mU/L)/(Glucose (mmol/L) 3.5)%.

### GLP-1R detection

Peripheral blood mononuclear cells (PBMCs) were harvested from the peripheral blood of male C57BL/6 J mice and cultured in 1640 complete medium at a concentration of 1 × 10^6^ cells/mL. Palmitate (PA), one of the most common saturated free fatty acid (FFA), is used to induce lipotoxic environment (Shao et al. 2014). After replacement of culture medium, the cells were cultured with or without 200 µM PA for 24 h. The expression of GLP-1R in PBMCs was detected using western blot.

### Culture of Th17 and Treg cells

Naïve CD4 + T cells were isolated from the spleens of male C57BL/6J mice using Naïve CD4 + T cells isolation kit (Miltenyi, Germany) (Au-Flaherty et al. 2015; Duan et al. 2019) and then cultured in 1640 medium containing 10% fetal bovine serum with plate-bound anti-CD3 (2 μg/mL, #14-0031-86, eBioscience, USA) and anti-CD28 (1 μg/mL, #14-0281-85, eBioscience, USA) antibodies for 4 days. IL-2 (200 U/mL, Cusabio, China) and TGF-β (0.5 ng/mL, Cusabio, China) were added to the culture medium for Treg cell differentiation; while IL-6 (10 ng/mL, Cusabio, China), IL-23 (10 ng/mL, Sinobiological, China) and TGF-β (2.5 ng/mL, Cusabio, China) were added to the culture medium for Th17 cell differentiation. After replacement of culture medium, cells were cultured with 200 µM PA and/or 100 nM exenatide in the presence or absence of 10 µM Foxrkhead box protein O1 (FoxO1) inhibitor (AS1842856, MedChemExpress, USA) for 24 h.

### Flow cytometry analysis

The frequencies of Th17 and Treg cells were investigated using flow cytometry. For Th17 assay, cells were stimulated with Cell Stimulation Cocktail (eBioscience, USA) for 6 h at 37 °C and 5% CO_2_ and then stained with anti-mouse CD4-phycoerythrin (PE) (#12-0041-82, eBioscience, USA). Fixation and permeabilization were performed with fix/perm buffer (Servicebio, China) and then cells were incubated with anti-mouse IL-17-allophycocyanin (APC) (#506915, eBioscience, USA). For Treg assay, cells were incubated with anti-mouse CD4-fluorescein isothiocyanate (FITC) (#11-0043-82, eBioscience, USA) and anti-mouse CD25-PE (#12-0251-81, eBioscience, USA) at 4 °C for 30 min in darkness. After fixation and permeabilization (Biolegend, USA), cells were stained with anti-mouse forkhead box P3 (Foxp3)-Alexa Fluor 647 (#126407, Biolegend, USA) at 4 °C for 30 min in darkness. Data collection and analysis were performed on a FACSCalibur (Beckman, USA).

### Quantitative real-time PCR

mRNA expression of FoxO1, IL-17 and Foxp3 was determined by real-time PCR. Total RNA was extracted using Trizol Reagent adhering to the manufacturer’s instructions (Scrvicebio, China). RNA concentration and purity were assessed using ultramicro spectrophotometer (NanoDrop2000, Thermo, USA). Afterwards, cDNA was generated from mRNA by using RevertAid First Strand cDNA Synthesis Kit (Thermo, USA). qRT-PCR was performed on Real-Time PCR System (ABI, USA), using FastStart Universal SYBR Green Master (Rox, Servicebio, China). The relative gene expression levels were calculated using the threshold cycle (CT), according to the 2^−∆∆CT^ method. GAPDH was set as reference. All RT-PCR specific primer sequences applied for PCR reaction amplification were presented in Additional file 1: Table S1.

### Western blot analysis

Protein samples were separated by SDS-PAGE and transferred to polyvinylidene fluoride membranes. After blocking with 5% non-fat milk in Tris-buffered saline Tween-20 (TBST), the membranes were incubated with primary antibodies against IL-17 (#AO688, Abclonal Technology, China), Foxp3 (#GB11093, Servicebio, China), phosphoinositide 3-kinase (PI3K) (#bsm-33219m, Bioss, China), phospho (p)-PI3K (Tyr317, #bs-5570R, Bioss, China), protein kinase B (Akt) (#GB11689, Servicebio, China), p-Akt (Ser473, #AF0908, Affinity, USA), FoxO1 (#GB11286, Servicebio, China), p-FoxO1 (Thr24, #9464T, Cell Signaling Technology, USA) or GLP-1R (#CSB-PA009514YA01HU, Cusabio, China) at 4℃ overnight. The membranes were then incubated with horseradish peroxidase-conjugated secondary antibodies. Proteins were detected using an enhanced chemiluminescence system (Clinx Science Instruments, China).

### Immunofluorescence staining

5 cm-thick paraffin sections of pancreatic tissues were subjected to immunofluorescence staining with antibodies against IL-17 (#GB11110, Servicebio, China) or Foxp3 (#GB11093, Servicebio, China) in combination with anti-insulin antibody (#GB12334, Servicebio, China) according to the manufacturer’s instructions. Nuclei were counterstained with DAPI stain (Servicebio, China). Immunostained images were acquired using Ortho-Fluorescent microscope imaging system (Nikon, Japan).

### HE staining

Pancreatic tissues were placed in 10% neutral formalin for fixation, embedded in paraffin blocks. After deparaffination and rehydration, 5-μm-thick paraffin sections were stained with hematoxylin and eosin (HE). Insulitis scoring was calculated according to the following criteria under light microscope: 0, no immune cell infiltration; 1, < 25% 25% of islet being infiltrated with immune cell; 2, 25–50%; 3, 50–75%; 4, > 75%. Data are presented as the percentage of islets with each grade of insulitis.

### Statistical analysis

All data were presented as mean ± standard error of the mean (SEM). Normality was tested by Kolmogorov–Smirnov. Comparisons were performed using t-test or ANOVA. All probability values were two-tailed, and *P* < 0.05 was considered significant.

## Results

### Exenatide improved body weight, insulin sensitivity and β-cell function

**Fig. 1:**
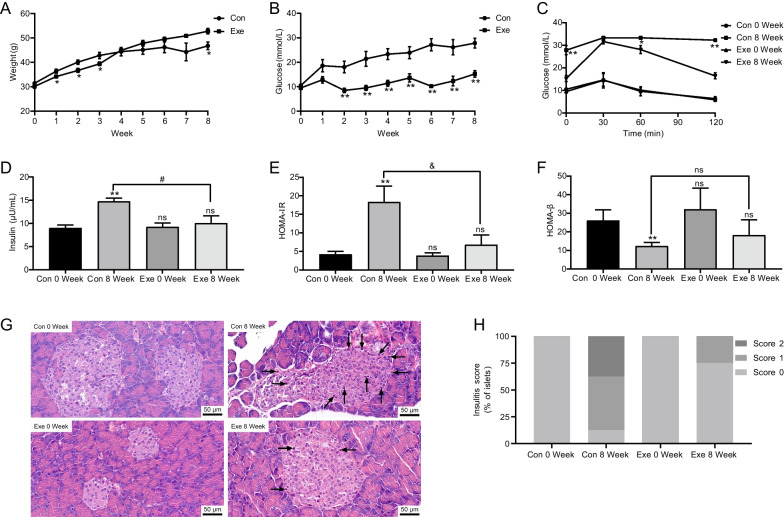
Time course changes of body weight (**A**) and blood glucose (**B**) in control (Con) and exenatide-treated (Exe) db/db mice. OGTT (**C**), fasting insulin (**D**), HOMA-IR (**E**), HOMA-β (**F**), representative HE micrographs of pancreatic insulitis (**G**), and the percentage of insulitis scores of pancreatic islets (**H**) in Con and Exe group before and after 8-week treatment. Data are presented as mean ± SEM, n = 8 in each group; **P < 0.01,*P < 0.05 vs control (**A**, **B**), control 8 week (**C**), or control 0 week (**D**–**F**); & *P* < 0.01, ^#^*P* < 0.05 vs control 8 week (**D**–**F**); ns *P* > 0.05. Scale bar = 50 μm. The black arrow indicates infiltrated cells. *OGTT* oral glucose tolerance test, *HOMA-IR* homeostasis model assessment of insulin resistance, *HOMA-β* homeostatic model assessment of β cell function

To determine the therapeutic effect of exenatide on T2DM, the time course changes in body weight and fasting blood glucose levels were monitored in db/db mice, the model of obese T2DM, treated with either saline solution or exenatide (Fig. 1 A,B). Compared with the control group, exenatide-treated mice showed significantly less weight gain within 3-week intervention (Exenatide 3 Week, 39.39 ± 0.73 g vs Control 3 Week, 42.81 ± 1.28 g, *P* = 0.048). However, such effect did not persist and the body weight in exenatide group gradually increased as compared with control group (Exenatide 8 Week, 52.74 ± 1.14 g vs Control 8 Week, 46.74 ± 1.54 g, *P* = 0.014). In addition, exenatide treatment could effectively control the glucose level in db/db mice and the fasting glucose ranged from 8.48 mmol/L to 15.22 mmol/L over the 8-week duration of the experiment (Exenatide 8 Week, 15.22 ± 1.35 mmol/L vs Control 8 Week, 27.82 ± 1.99 mmol/L, *P* < 0.001).

Moreover, OGTT study showed significant improvement of glucose tolerance after 8-week exenatide treatment as shown in Fig. 1C. In addition, at the end of the study, exenatide group exhibited obviously lower levels of fasting insulin than control group (Exenatide 8 Week, 10.06 ± 1.57 µU/mL vs Control 8 Week, 14.72 ± 0.75 µU/mL, *P* = 0.028, Fig. 1D). Furthermore, HOMA-IR and HOMA-β were calculated and it was demonstrated that exenatide intervention could markedly improve either insulin sensitivity (Exenatide 8 Week, 6.80 ± 1.18 vs Control 8 Week, 18.34 ± 1.92, *P* < 0.001, Fig. 1E) or β-cell function (Exenatide 8 Week, 18.20 ± 3.69 vs Control 8 Week, 12.33 ± 0.86, *P* > 0.05, Fig. 1F).

### Exenatide alleviated the severity of islet inflammation with the progression of T2DM

We next investigated the effect of exenatide on islet inflammation. According to the HE staining, the percentage of islets with immune cell infiltration was slightly increased in control diabetic mice at week 8 (Control 8 Week, 87.5% vs Control 0 Week, 0%, Fig. 1G, H), indicating low-grade inflammatory state in pancreatic islets as the development of T2DM. On the contrary, the inflammatory state was alleviated in a certain degree after 8-week treatment of exenatide (Exenatide 8 Week, 25% vs Control 8 Week, 87.5%, Fig. 1G, H), indicating the potential effect of GLP-1 on the regulation of inflammation state in pancreatic islet.

### Exenatide restored periphery Th17/Treg balance in db/db mice

**Fig. 2:**
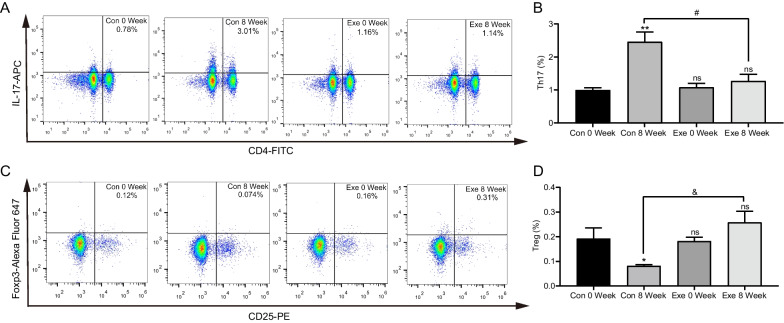
Peripheral frequency of Th17/Treg in db/db mice via flow cytometry. **A** Representative flow cytometric plots of Th17 cells from control (Con) and exenatide (Exe) groups at week 0 and week 8. **B** Statistical analysis of the percentage of Th17 cells in control and exenatide groups. **C** Representative flow cytometric plots of Treg cells with CD4-FITC, CD25-PE and Foxp3 staining. **D** Statistical analysis of the percentage of Treg cells. Data are presented as mean ± SEM; n = 8 in each group; **P < 0.01, *P < 0.05 vs control 0 week; & *P* < 0.01, # *P* < 0.05 vs control 8 week; ns *P* > 0.05

To further investigate the systemic immunoregulatory effects of exenatide, we detected the frequencies of Th17 and Treg cells in the peripheral blood of db/db mice by flow cytometry. Along with the progression of diabetes, we found that the proportion of CD4 + IL-17 + Th17 cells was increased from 0.99 ± 0.07% to 2.46 ± 0.30% after 8-week treatment (*P* = 0.001, Fig. 2A, B) while CD4 + CD25 + Foxp3 + Treg cells were markedly decreased (Control 8 Week, 0.08 ± 0.01% vs Control 0 Week, 0.19 ± 0.04%, *P* = 0.037, Fig. 2C, D). These data further verified the imbalance of Th17/Treg as the development of T2DM. Of note, it was demonstrated that there was no significant difference of Th17 frequency in exenatide group before and after 8-week treatment (Fig. 2A, B), indicating that exenatide intervention could block the increase of peripheral Th17. Similar results were found for Treg cells (Fig. 2C, D). Taken together, these results disclosed that exenatide may exert its systemic immuno-inflammatory regulation effect in obese diabetic mice through correcting the imbalance of Th17/Treg cells.

### Exenatide prevented Th17 cells from infiltrating into islets

**Fig. 3:**
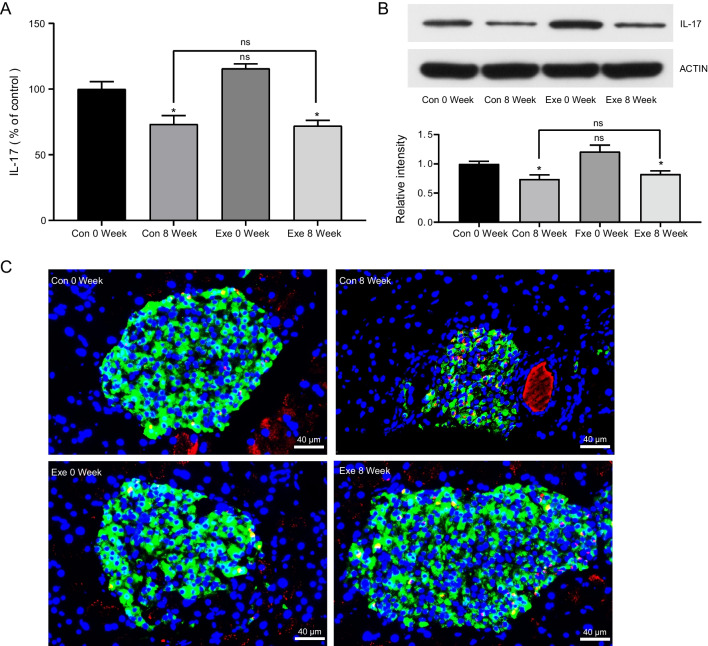
Detection of Th17 cells in pancreas from control (Con) and exenatide (Exe) groups at week 0 and week 8. **A** mRNA levels of IL-17 by real-time PCR. mRNA quantities were calculated as a ratio to the level of GAPDH mRNA in each sample. Data are shown as the relative expression ratio to Control 0 Week. Results are expressed as mean ± SEM, n = 8 in each group. **B** Representative image and gray value analysis of IL-17 by western blot. ACTIN was used as a loading control. Data are shown as the relative expression ratio to Control 0 Week and expressed as mean ± SEM, n = 8 in each group. **C** Representative immunofluorescence of pancreatic sections using anti-IL-17 staining (red), anti-insulin staining (green) and DAPI staining (blue), scale bar = 40 μm, n = 8 in each group. *P < 0.05 vs control 0 week; ns *P* > 0.05

To examine whether Th17/Treg imbalance also occurred in pancreas in obese T2DM, we detected the expression of IL-17 and Foxp3 in pancreas by real-time PCR and western blot. Surprisingly, both the mRNA and protein levels of IL-17 were decreased significantly as the progression of T2DM (Fig. 3A, B), which was inconsistent with the findings in peripheral blood. In addition, exenatide intervention had no impact on the mRNA expression of IL-17 (Exenatide 8 Week, 71.96 ± 4.28% vs Control 8 Week, 73.17 ± 6.68%, *P* > 0.05, Fig. 3A). Similar results were shown in western blot detection (Exenatide 8 Week, 0.83 ± 0.06 vs Control 8 Week, 0.74 ± 0.07, *P* > 0.05, Fig. 3B). We further detected the expression of IL-17 and Foxp3 via immunofluorescence analysis. The findings demonstrated that, as the development of T2DM, IL-17 + T cells gradually migrated from the periphery of pancreas islet to the center of the islet; while Th17 cells maintained to infiltrate surrounding the islet in exenatide-treated mice (Fig. 3C), disclosing that exenatide treatment could change the distribution rather than the proportion of IL-17 + T cells in pancreas. Of note, neither PCR nor western could detect the expression of Foxp3 in pancreas (Data not shown). Foxp3 + Treg cells were undetectable in pancreas by immunofluorescence staining as well. Accordingly, it is speculated that GLP-1 may exert its protective effect on islet inflammation and β-cell function, partly, through preventing Th17 cells infiltrating into pancreatic islets rather than modulating their frequencies.

### Exenatide regulated the proliferation of Th17 and Treg cells in vitro while FoxO1 inhibitor blocked this effect

**Fig. 4:**
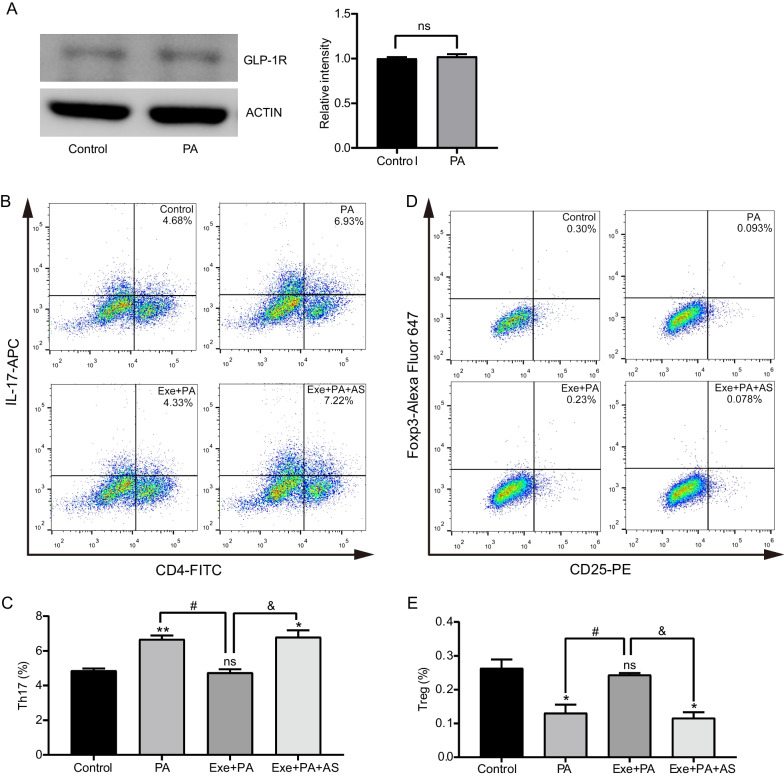
Effects of exenatide on Th17/Treg proliferation in vitro. **A** The expression of GLP-1R in PBMCs from C57BL/6 J mice by western blot. Representative image and gray value analysis of GLP-1R from Control and palmitate (PA) groups. Data are presented as mean ± SEM of three independent experiments. ACTIN was used as a loading control. Naïve CD4 + T cells collected from C57BL/6 J mice were cultured under Th17/Treg-inducing conditions with palmitate (PA), exenatide plus palmitate (Exe + PA) and exenatide, palmitate plus FxoO1 inhibitor AS1842856 (Exe + PA + AS). **B** Representative flow cytometric plots of Th17 cells from different groups. **C** Statistical analysis of the percentage of Th17 cells in different groups. **D** Representative flow cytometric plots of Treg cells with CD4-FITC, CD25-PE and Foxp3 staining. **E** Statistical analysis of the percentage of Treg cells. Data are presented as mean ± SEM of three independent experiments. **P < 0.01, *P < 0.05 vs control group; ^#^*P* < 0.05 vs PA group; & *P* < 0.05 vs Exe + PA group; ns *P* > 0.05

The expression of GLP-1R in PBMCs of C57BL/6 J mice could be identified by western blot (Fig. 4A). Notably, the levels of GLP-1R showed no significant difference between control and PA groups (*P* > 0.05, Fig. 4A), indicating that PA had little effect on the expression of GLP-1R.

To further verify the effect of GLP-1 in regulating the proliferation of Th17 and Treg cells, Naïve CD4 + T cells from the spleens of C57BL/6 J mice were collected. Since lipotoxicity is the most critical condition in diabetes with obesity and induces a chronic low-level inflammation in metabolic tissues (Longo et al. 2019), the percentages of Th17 and Treg cells were detected with the administration of PA and exenatide. It was found that PA administration significantly increased the proportion of Th17 cells (PA, 6.67 ± 0.22% vs Control, 4.86 ± 0.12%, *P* = 0.004, Fig. 4B, C) while decreased the percentage of Treg cells (PA, 0.13 ± 0.02% vs Control, 0.26% ± 0.03%, *P* = 0.039, Fig. 4D, E). Significantly, the treatment of exenatide corrected palmitate induced changes of both Th17 (Exenatide + PA, 4.75 ± 0.20% vs PA, 6.67 ± 0.22%, *P* = 0.006, Fig. 4B, C) and Treg (Exenatide + PA, 0.24 ± 0.01% vs PA, 0.13 ± 0.02%, *P* = 0.021, Fig. 4 D,E). The supplementation of FoxO1 inhibitor AS1842856 blocked the regulating effect of exenatide on both Th17 (Exenatide + PA + AS, 6.80 ± 0.38% vs PA, 6.67 ± 0.22%, *P* > 0.05, Fig. 4B, C) and Treg (Exenatide + PA + AS, 0.12 ± 0.02% vs PA, 0.13 ± 0.02%, *P* > 0.05, Fig. 4D, E).

Taken together, these findings verified that exenatide treatment could correct Th17/Treg imbalance under lipotoxic stress. In addition, such effect could be blocked when FoxO1 was inhibited, disclosing that FoxO1 signal pathway may be involved in.

### Exenatide regulated Th17/Treg proliferation via the PI3K/Akt/FoxO1 pathway

**Fig. 5:**
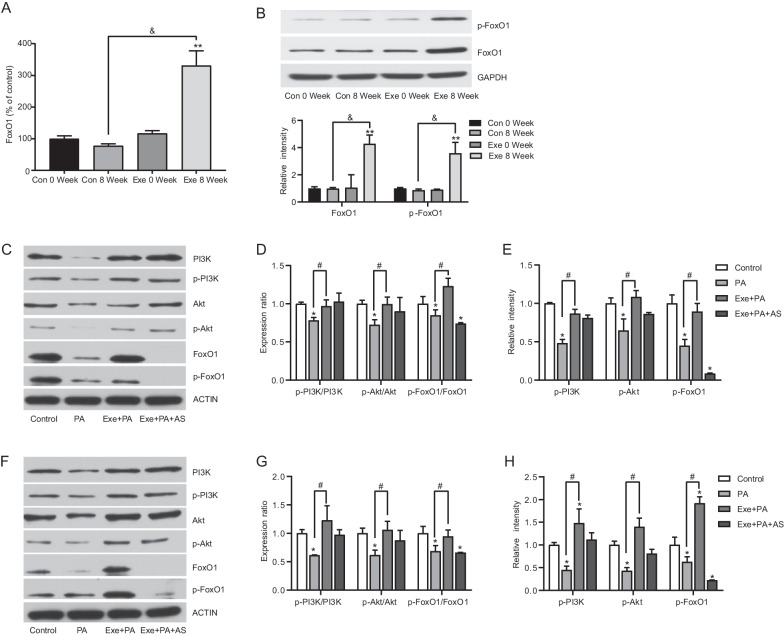
Effects of exenatide on Th17/Treg proliferation via PI3K/Akt/FoxO1 signaling. **A** mRNA levels of FoxO1 in PBMCs from control (Con) and exenatide (Exe) groups detected by real-time PCR. mRNA quantities were calculated as a ratio to the level of GAPDH mRNA in each sample. Data are shown as the relative expression ratio to Con 0 Week. Results are expressed as mean ± SEM, n = 8 in each group. **B** Representative image and gray value analysis of Foxo1 and phosphor (p)-FoxO1 in PBMCs from Con and Exe groups detected by western blot. ACTIN was used as a loading control. Data are shown as the relative expression ratio to Con 0 Week and expressed as mean ± SEM, n = 8 in each group. **C** Th17 cells were treated with palmitate (PA), exenatide plus palmitate (Exe + PA) and exenatide, palmitate plus FxoO1 inhibitor AS1842856 (Exe + PA + AS). Representative image of total PI3K, Akt, FoxO1 and p-PI3K, p-Akt, p-FoxO1 in Th17 cells in vitro detected by western blot. **D** Gray value analysis of p-PI3K/PI3K, p-Akt/Akt and p-FoxO1/FoxO1 in Th17 cells. **E** Gray value analysis of p-PI3K, p-Akt and p-FoxO1 in Th17 cells. **F** Treg cells were treated with PA, Exe + PA and Exe + PA + AS. Representative image of total PI3K, Akt, FoxO1 and p-PI3K, p-Akt, p-FoxO1 in Treg cells in vitro detected by western blot. **G** Gray value analysis of p-PI3K/PI3K, p-Akt/Akt and p-FoxO1/FoxO1 in Treg cells. **H** Gray value analysis of p-PI3K, p-Akt and p-FoxO1 in Treg cells. Data are shown as the relative expression ratio to Control. Three independent experiments were performed and results are expressed as mean ± SEM. **P < 0.01, *P < 0.05 vs control 0 week (**A**, **B**), or control group (**D**, **E**, **G**, **H**); & *P* < 0.01, ^#^*P* < 0.05 vs control 8 week (**A**, **B**), or PA group (**D**, **E**, **G**, **H**); ns *P* > 0.05

To explore whether exenatide-modulated proliferation of Th17/Treg was mediated by FoxO1 signal pathway, the expression levels of FoxO1 and p-FoxO1 were measured in vivo and in vitro. Our findings demonstrated that mRNA level of FoxO1 in PBMCs from exenatide-treated db/db mice increased significantly at the end of study by 230.59% compared with Control 0 Week (Fig. 5A). Protein levels of FoxO1 and p-FoxO1 exhibited the similar changes (Fig. 5B), suggesting that FoxO1 might be a target of exenatide in regulating Th17 and Treg cells. Furthermore, our previous study has estimated that protective effect of GLP-1 was mediated by PI3K/Akt/FoxO1 signaling pathway (Shao et al. 2014). Therefore, we detected the levels of PI3K/Akt/FoxO1 and their respective phosphorylated forms in Th17 and Treg cells in vitro. It was found that palmitate exposure decreased PI3K, Akt, FoxO1 and their phosphorylation levels compared with control group in Th17 cells (Fig. 5C–E). And such decrease could be completely corrected by exenatide intervention. Similar results were observed in Treg cells as well (Fig. 5F–H). Taken together, these findings suggested that exenatide suppressed the differentiation of Th17 cells while promoted Treg development, which may be partly mediated by PI3K/Akt/FoxO1 pathway.

## Discussion

The recognition of chronic low-grade inflammation in the development of obese T2DM has been paid increasing attention. Our previous study has discussed that Th17/Treg imbalance may be involved in this inflammatory process (Wang et al. 2018). Although exenatide was widely applied as hypoglycemic agent in T2DM, its immuno-inflammatory regulation properties are being recognized gradually (Alicic et al. 2021; Lee et al. 2016). However, studies on the effect and underlying mechanisms of exenatide on Th17/Treg balance in T2DM are limited. Our present study demonstrated that exenatide significantly suppressed the proportion of Th17 cells but enhanced Treg cells in vivo and in vitro, which may be mediated by PI3K/Akt/FoxO1 pathway.

It is known that function of pancreatic β cells is gradually impaired by excessive glucose (glucotoxicity) and fatty acids (lipotoxicity) under the context of obese T2DM (Shao et al. 2014). Additionally, obesity-associated systemic low-grade inflammation could cause an elevation of inflammatory cytokines and chemokines in circulation together with the accumulation and impaired function of various immune cells including macrophages and B cells in pancreas, resulting in islet inflammation and β-cell dysfunction (Eguchi et al. 2017; Guo et al. 2020; Ying et al. 2019). Consistently, our current study observed decreased HOMA-β and increased insulitis as the progress of diabetes in db/db mice (Fig. 1), further strengthening the viewpoint that islet inflammation may be involved in β-cell dysfunction in obese T2DM. Recently, T cells-mediated immunity was identified to be intertwined with metabolic disorders (Lee et al. 2018). Dalmas et al. reported that an increased peripheral frequency of effector T cells (Teffs) promoted glycemic deterioration in obese T2DM patients (Dalmas et al. 2014); while an increase in Treg frequencies restored insulin sensitivity (Eller et al. 2011). In this study, we demonstrated that the peripheral frequency of Th17 cells increased but Treg proportion reduced markedly following the development of diabetes (Fig. 2), which is consistent with data from clinical studies conducted in obese or T2DM patients (Ip et al. 2016; Yuan et al. 2017). These findings disclose a clue that the peripheral imbalance of Th17/Treg and the resultant systemic inflammatory status may contribute to the β-cell dysfunction and the progression of T2DM.

Furthermore, infiltration of Th17 cells in pancreas was validated in non-obese diabetic (NOD) mice, a T1DM mouse model characterized by spontaneous progressive insulitis and severe hyperglycemia. Such infiltration was associated with the pathogenesis of T1DM (Bellemore et al. 2016; Martin-Orozco et al. 2009). A previous study from Grieco and colleagues have demonstrated that Th17 immunity aggravated insulitis by inducing β-cell apoptosis and exacerbating chemokines expression; the latter may in turn augment the attraction of invading immune cells in T1DM (Grieco et al. 2014). Similarly, Honkanen et al. also demonstrated that IL-17 inhibited the mRNA expression of antiapoptotic gene and enhanced cytokines-induced proapoptotic effects in human islet cells (Honkanen et al. 2010). However, there is no research that investigate the infiltration of Th17 and Treg in pancreas under the context of obese T2DM. Of note, our study identified the migration of Th17 cells from the islet periphery into intra-islet, which may contribute to the insulitis and β cell failure. Interestingly, the present study identified that the level of IL-17 expression in pancreas was decreased as the progression of T2DM, which was contradictory to the findings of circulating Th17 cells. This may suggest tissue-specific immunity during disease progression. Cavallari et al. identified that cytokines related to Th17 responses decreased in the gut but increased in the liver during obesity (Cavallari et al. 2016). Thus, we assume that there is compartmentalization of Th17 immune responses in different tissues under the context of T2DM, which needs to be further elucidated. Furthermore, it is recognized that Th17 cells could switch to other T helper cell subsets under certain inflammatory milieu (Martin-Orozco et al. 2009; Stadhouders et al. 2018), which may explain the decreased level of IL-17 + cells in pancreas identified in this study. Martin-Orozco et al. reported that Th17 cells could convert into interferon-γ (IFN-γ) producing Th1 cells to promote β-cell apoptosis and pancreatic inflammation in NOD mice (Martin-Orozco et al. 2009). Accordingly, we assume that the Th17 transdifferentiation and plasticity within pancreas may also contribute to the inflammatory responses and the development of T2DM.

In the current study, Foxp3 + Tregs were undetectable in pancreas. Similarly, Willcox and colleagues analyzed postmortem pancreatic samples from 16 T1DM patients, with Foxp3 + Tregs detectable only in a single patient (Willcox et al. 2009). Study from Nti et al. also reported that the number of Tregs were decreased to undetectable levels in the pancreatic lymph nodes of the untreated hyperglycemic NOD mice (Nti et al. 2012). Deficiency of Tregs may result in the progress of inflammation and diabetes. A previous study from Watts et al. reported that depletion of Foxp3 + Tregs precipitates destructive β-cell autoimmunity in NOD.DEREG (‘depletion of regulatory T cell’) mouse model (Watts et al. 2021). Conversely, transfer of Tregs could largely prevent Teffs-induced diabetes development in NOD mice (Sprouse et al. 2018). Accordingly, these data suggested that the lack of Treg cells may be one of the possible causes for the development of islet inflammation in T2DM.

Nowadays, GLP-1RA is widely implemented in therapy for weight control and T2DM (Meier 2012). The weight loss effect of short-term exenatide has been widely demonstrated in different animal models and clinical conditions. Of note, in our study, although less body weight gain was observed in exenatide-treated mice, such effect could only persist for 3 weeks (Fig. 1). Consistently, Mack et al. also reported that 4-week exenatide treatment significantly reduced body weight in high-fat-fed rodents (Mack et al. 2006). On the contrary, a recent study found that, although exenatide intervention could improve lipid deposition and insulin sensitivity in ob/ob mice (leptin-deficient obese T2DM mice model), it failed to reduce body weight (Xu et al. 2020). In addition, a 12-week randomized, single-blind study reported that exenatide treatment could only achieved modest weight loss in less than half of participants (Rodgers et al. 2021). Indeed, the effect of weight loss in response to exenatide treatment is varied under different clinical conditions and the reason needs to be further elucidated (Dushay et al. 2012).

Moreover, in vitro, in vivo, and clinical studies over the last decades have collectively demonstrated that GLP-1 and GLP-1RAs have beneficiary effects on preservation of β-cell function. Our results showed that exenatide intervention remarkably ameliorated HOMA-β, which confirmed its protective action on β-cell function. It is considered that weight loss could contribute to the recovery of β-cell function (Taylor et al. 2018). However, the effect of weight control by exenatide in our study could only be preserved for 3 weeks. Thus, we assumed that the preservation of β-cell function by short-term exenatide intervention may be partially independent of the weight loss (Shao et al. 2014).

There are various direct protective effects of GLP-1 on pancreatic β-cell function, including inhibition of glucolipotoxic ER stress, regulation of transcription factors and signaling molecules that is implicated in β-cell proliferation, and prevention of cell apoptosis mediated by the induction of anti-apoptotic proteins such as Bcl-2 and Bcl-xl (Lee et al. 2014). Interestingly, beyond these mechanisms, the anti-inflammatory properties of GLP-1RA have been gradually identified (Lee et al. 2016), which lies on the wide distribution of GLP-1Rs in various immune cells (Hadjiyanni et al. 2010; Hogan et al. 2011; Stahle et al. 2021; Tanaka et al. 2016). Likewise, we also identified the expression of GLP-1R in PBMCs. In addition, lipotoxic condition did not affect the level of GLP-1R expression.

A handful of preclinical studies disclosed that GLP-1RA administration could affect systemic inflammatory status by suppressing the secretion of IL-17 in the serum of high-fat-fed mice (Sha et al. 2019) and increasing the level of serum TGF-β in NOD mice (Gao et al. 2021). In addition, the production of pro-inflammatory cytokines including tumor necrosis factor (TNF)-α, IL-1β and IL-6 in the peripheral blood of T2DM patients was found to be decreased obviously after the administration of exendin-4 (He et al. 2013). All these findings indicated the potential of GLP-1 on immune-modulation although some researchers consider these effects may be attributed to its metabolic benefits. The underlying mechanisms of GLP-1 on direct immuno-inflammatory regulation are not quite understood. Most of related studies were performed in autoimmune diseases. Moschovaki et al. demonstrated that GLP-1RA could protect mice from a nondiabetic, T-cell-dependent glomerulonephritis model by inhibiting the renal infiltration macrophages, CD8 + cytotoxic T cells and CD4 + T cells (Moschovaki Filippidou et al. 2020). A study from Chiou and colleagues considered that GLP-1RA could modulate the differentiation of Th1/Th17, providing mechanistic insight on T cells regulation in ameliorating experimental autoimmune encephalomyelitis by GLP-1 (Chiou et al. 2019). Furthermore, co-culture with exenatide reduced the levels of IL-1β, IL-2, IL-17 and IFN-γ in human islet supernatants (Cechin et al. 2012), disclosing that Th17 cell may be involved in GLP-1 related immuno-inflammatory modulation under diabetes context as well. On the other hand, GLP-1RAs are found to increase the frequency of Tregs in NOD mice (Xue et al. 2008) and high-fat-diet-induced obesity mice (Sha et al. 2019). Consistently, our findings demonstrated that the imbalance of Th17/Treg in peripheral blood were obliterated by exenatide supplementation under the context of obese T2DM. Accordingly, it is speculated that the restoration of peripheral Th17/Treg balance by exenatide contributes to the alleviation of systemic inflammatory status, resulting the improvement of glucose homeostasis, insulin sensitivity and β-cell function.

Inconsistent with the findings from peripheral blood, the frequency of IL17 + T cells in pancreas showed insignificant difference between control and exenatide-treated mice. According to immunofluorescence analysis, at the end of the study, IL17 + T cells mainly invaded in the center of the islets in controls, but gathered around peri-islet in exenatide group. It seems that exenatide treatment prevented or attenuated the infiltration of IL-17 + Th17 cells into pancreatic islets rather than altering the level of Th17 cells. It has been demonstrated that migration and infiltration of immunocytes into inflamed islets are an essential component of the immune response of β-cell destruction (Khodabandehloo et al. 2016). In this regard, exenatide is assumed to prevent the development of destructive insulitis, partially through inhibiting intra-islet infiltration of Th17 cells. It is recognized that IL-17 + T cells are expressed chemokine (CC motif) receptor 6 (CCR6) (Honkanen et al. 2010), which plays an important role in migration and infiltration of Th17 cells at inflamed tissues (Singh et al. 2014). A study from Lee and colleagues demonstrated that resveratrol treatment reduced the severity of insulitis by inhibiting CCR6-chemokine (C–C motif) ligand 20 (CCL20)-mediated Th17 cell migration from peripheral lymphoid organs to pancreas (Lee et al. 2011). Furthermore, Bang-Berthelsen et al. reported that GLP-1RA liraglutide could alleviate colonic inflammation partially by downregulating CCL20 levels in a colitis mouse model (Bang-Berthelsen et al. 2016). Therefore, exenatide may block Th17 migration into islet by regulating Th17-associated chemokines, which needs to be further evidenced in our future study.

However, Foxp3 + Tregs were still undetectable in pancreas in exenatide-treated group. To the best of our knowledge, there is no similar study regarding to the effects of GLP-1RAs on the Treg frequency and function in pancreas. Previous study found that exendin-4 could cause an increasing trend of Treg number in lymph nodes of NOD mice, although such change was insignificant (Drucker et al. 2008). It is speculated that the immune-regulatory effects of exenatide on the promotion of Tregs may be weak in pancreas of T2DM. Larger dose and longer intervention of exenatide may make the effect obvious.

The PI3K/Akt pathway is essential for the development of T cells (Juntilla et al. 2008) and is often dysregulated in various inflammatory disorders (Li et al. 2020; Stylianou et al. 2011). One mechanism by which PI3K/Akt regulate T cell differentiation is through the modulation of FoxO1 (Hedrick et al. 2012). FoxO1 is critical for the differentiation and function of Tregs by up-regulating the activity of Foxp3 promoter (Ouyang et al. 2010). In addition, it could act as a potent anti-inflammatory control switch through the suppression of ROR-γT-induced Th17 differentiation program (Laine et al. 2015). Although various studies have highlighted the specific role of FoxO1 in T cell biology (Hedrick et al. 2012), it remains unclear whether FoxO1 presides the regulation of Th17/Treg differentiation in the context of diabetes. In the current study, a decreasing tendency of FoxO1 was observed along with elevated Th17 cells and reduced Tregs following the progress of diabetes in db/db mice. In addition, declined levels of p-PI3K, p-Akt, and p-FoxO1 were observed under lipotoxic stress in vitro. Taken together, these findings disclose that PI3K/Akt/FoxO1 pathway may be involved in the differentiation and proliferation of Th17 and Treg cells under the context of T2DM and obesity.

The regulatory role of GLP-1RAs mediated by PI3K/Akt/FoxO1 signaling has been implicated in multiple disease process (Chen et al. 2019; Shao et al. 2014). Our previous study has demonstrated that liraglutide protected β-cell function under lipotoxic stress via PI3K/Akt/FoxO1 pathway (Shao et al. 2014). In the current study, the decreased phosphorylation of PI3K/Akt/FoxO1 could be completely canceled by exenatide intervention accompanied by the restoration of Th17/Treg balance. Moreover, all the effects of exenatide on CD4 + T cells proliferation were prohibited when AS1842856 was pre-incubated. All these findings disclose that exenatide may regulate the differentiation of Th17 and Treg cells through PI3K/Akt/FoxO1 pathway.

There are a couple of limitations in this study. The intervention duration of GLP-1RA treatment was 8 weeks according to our previous research (Shao et al. 2014). Dynamic observation of Treg and Th17 cells in pancreas at different time intervals would be of significance. Additionally, other mechanisms may be involved in GLP-1RA regulated islet inflammation and β-cell function. Velmurugan et al. reported that exendin-4 intervention could suppress the expression of inflammatory genes such as nuclear factor kappa-B1 (NFκB1), NFκB2 and TNF receptor superfamily member 1A in cultured human islets (Velmurugan et al. 2012). Effects of anti-oxidative stress and anti-ER stress by GLP-1RA in β cells (Kim et al. 2010; Oh et al. 2013) are likely interconnected with the improvement of β-cell function. Co-cultures of Th17/Treg-β cell in vitro help to provide more direct evidences.

## Conclusions

Our results indicate that the preservation of β-cell function by exenatide treatment may be mediated by alleviating both systemic inflammation via restoration of peripheral Th17/Treg balance and islet inflammation through inhibiting intra-islet infiltration of Th17 cells. In addition, PI3K/Akt/FoxO1 pathway was involved in the regulation of Th17/Treg balance, disclosing a potential mechanism exenatide-related immuno-inflammatory modulation in obese T2DM.

## Supplementary Information

**Additional file 1: Table S1.** List of primers used for qRT-PCR analysis.

## Data Availability

The datasets used and/or analyzed during the current study are available from the corresponding author on reasonable request.

## Abbreviations

Akt: Protein kinase B
cAMP: Cyclic adenosine monophosphate
CCR6: Chemokine (CC motif) receptor 6
CCL20: Chemokine (C–C motif) ligand 20
ER: Endoplasmic reticulum
FFA: Free fatty acid
FoxO1: Foxrkhead box protein O1
Foxp3: Forkhead box P3
HE: Hematoxylin and eosin
GLP-1: Glucagon-like peptide-1
GLP-1R: Glucagon-like peptide-1 receptor
GLP-1RA: Glucagon-like peptide-1 receptor agonist
HOMA-β: Homoeostasis model assessment of β-cell function
HOMA-IR: Homoeostasis model assessment of insulin resistance
IL: Interleukin
IFN-γ: Interferon-γ
LPS: Lipopolysaccharide
NFκB: Nuclear factor kappa-B
NOD: Non-obese diabetic
NTS: Nephrotoxic serum nephritis
OGTT: Oral glucose tolerance test
PA: Palmitate
PBMCs: Peripheral blood mononuclear cells
PI3K: Phosphoinositide 3-kinase
ROR-γt: Retinoid-related orphan receptor gamma-t
T1DM: Type 1 diabetes mellitus
T2DM: Type 2 diabetes mellitus
Teffs: Effector T cells
TGF-β: Transforming growth factor-β
Th17 cell: T helper 17 cell
TLR4: Toll-like receptor 4
TNF: Tumor necrosis factor
Treg cell: Regulatory T cell
