# Supplementary material for: Exenatide regulates Th17/Treg balance via PI3K/Akt/FoxO1 pathway in db/db mice
Source: Mol Med. 2022 Dec 3;28:144. doi: 10.1186/s10020-022-00574-6 (PMC9719171; doi:10.1186/s10020-022-00574-6)
Supplement: Supplementary file 1 — Additional file 1: Table S1. List of primers used for qRT-PCR analysis. [file 10020_2022_574_MOESM1_ESM.doc]

**Supplemental materials**

**Table S1 List of primers used for qRT-PCR analysis.**

| Gene | Primer Sequence (5′-3′) |
| --- | --- |
| Mouse IL-17 | F: CTGTGTCTCTGATGCTGTTGCTG  R: CGTGGAACGGTTGAGGTAGTC |
| Mouse Foxp3 | F: TTCACCTATGCCACCCTTATCC  R: GCTCCTCTTCTTGCGAAACTCA |
| Mouse FoxO1 | F: AAGGCCATCGAGAGCTCAGC  R: GATTTTCCGCTCTTGCCTCC |
| Mouse GAPDH | F: CCTCGTCCCGTAGACAAAATG  R: TGAGGTCAATGAAGGGGTCGT |

Note: F, Forward; FoxO1, Forkhead box O1; Foxp3, Forkhead box P3; R, Reverse
